# Synthesis and Characterization of Li_4_(OH)_3_Br for Thermal Energy Storage

**DOI:** 10.1021/acsaem.5c00359

**Published:** 2025-04-04

**Authors:** Emily Milan, James A. Quirk, John Cattermull, Andrew L. Goodwin, James A. Dawson, Mauro Pasta

**Affiliations:** † Department of Materials, University of Oxford, Oxford OX1 3PH, U.K.; ‡ Chemistry—School of Natural and Environmental Sciences, Newcastle University, Newcastle upon Tyne NE1 7RU, U.K.; § Department of Chemistry, University of Oxford, Oxford OX1 3QR, U.K.

**Keywords:** phase change material, peritectic compound, crystal structure, enthalpy, storage capacity

## Abstract

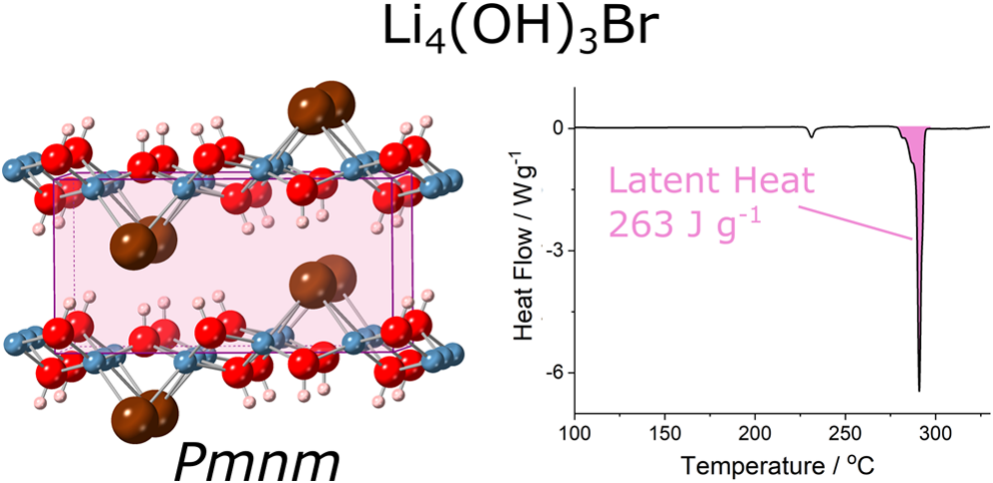

The peritectic compound Li_4_(OH)_3_Br has been suggested as a candidate material for latent heat thermal energy storage (TES), due to its high calculated melting enthalpy (804 J g^–1^) around 300 °C, however experimental reports have obtained much lower values (≤250 J g^–1^). In this work, we show that the crystal structure established for Li_4_(OH)_3_Br in literature corresponds to a metastable hydrated compound, and instead propose that the thermodynamically stable phase belongs to the *Pmnm* space group. The hydrated phase dehydrates at ∼175 °C, rendering the exceptional previous predictions inapplicable. An experimentally measured melting enthalpy of 263 ± 3 J g^–1^ is found for high-purity Li_4_(OH)_3_Br. Theoretical modeling is used to suggest a crystal structure for Li_4_(OH)_3_Br, from which a melting enthalpy of 260 J g^–1^ is calculated, in good agreement with the experimental work, and supporting that nonetheless impressive storage capacity at ∼290 °C can be offered by Li_4_(OH)_3_Br.

## Introduction

Increasing demand for efficient energy storage solutions has stimulated research into thermal energy storage (TES), particularly in the context of renewable energy integration and waste heat recovery. Employing TES for the capture and retention of excess thermal energy enables its use during periods of high demand or low energy production. Currently TES systems can be classified into 3 categories: sensible heat, latent heat, and thermochemical energy storage.^1^ Among these, latent heat storage, which exploits the thermal energy associated with phase changes, is gaining increasing attention for the high energy densities offered and the potential for efficient thermal regulation.^1^

Latent heat thermal energy storage (LHTES) systems store energy during the phase transition of a material, most commonly between solid and liquid phases, allowing for significant energy storage without a substantial change in temperature.^1^ Consistent output temperatures are crucial for power generation applications, such as concentration solar plants (CSP), where systems are optimized to operate at a specific temperature, with higher temperatures resulting in a more efficient thermodynamic cycle. Additionally, maintaining constant temperatures reduces thermal stress on the system’s components, helping to prolong their lifetime.

Various phase-change material (PCM) systems have been explored for high-temperature LHTES. Inorganic materials such as molten salts (e.g., NaNO_3_, KNO_3_) and eutectic mixtures (e.g., NaCl-KCl, NaNO_3_–KNO_3_) are prominent due to their high latent heat capacity, stability at elevated temperatures, and relatively low costs.^2−4^ However, challenges arise regarding corrosion, low thermal conductivity, and phase segregation.

In 2017, Achchaq considered the use of peritectic compounds for application as PCMs, conducting an extensive search of 635 binary systems in the temperature range 300 to 700 °C using FactSage software.^5,6^ Calculations found an impressive transition enthalpy as high as 804 J g^–1^ (425 kW h m^–3^) for the peritectic formation of Li_4_(OH)_3_Br at ∼300 °C. By comparison, steam accumulators, which are the predominant technology currently used in industry for the temperature range 250 to 300 °C, exhibit low storage capacities of 20–30 kW h m^–3^.^7,8^ This, and the relatively low cost of LiBr and LiOH salts, makes the use of Li_4_(OH)_3_Br in TES applications very appealing, particularly for use in direct steam generation (DSG) concentration solar plants (CSP) where isothermal storage capacity at 300 °C is desirable.^9^ Consequently, Li_4_(OH)_3_Br has attracted attention in research for potential application in latent heat storage (LHS).^8,10−14^

**Figure 1:**
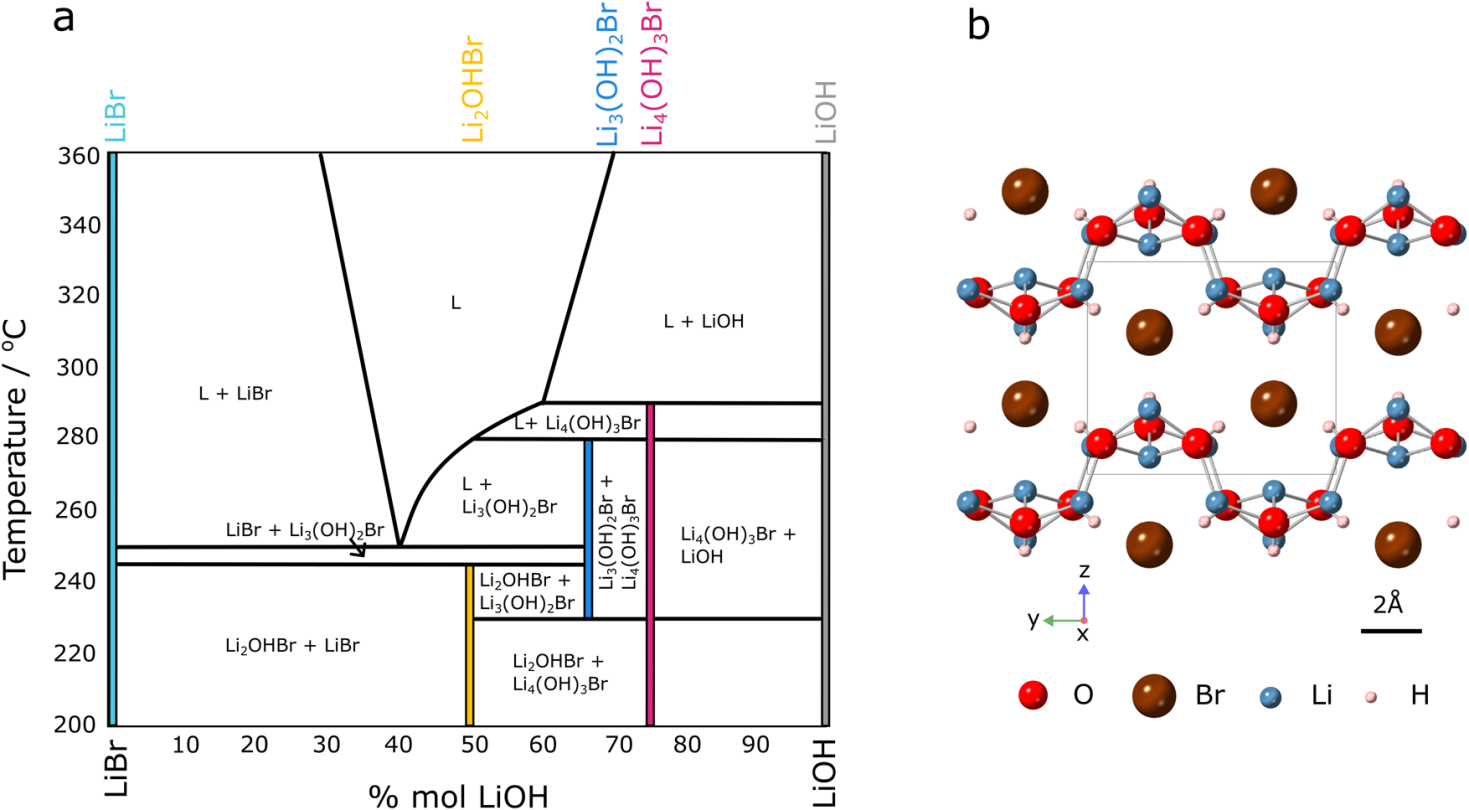
Existing Literature. (a) A drawing of the LiBr-LiOH phase diagram proposed by Mahroug et al.,^8^ adapted from [25]. Copyright 2025 American Chemical Society. Stoichiometric compounds LiBr, Li_2_OHBr, Li_3_(OH)_2_Br, Li_4_(OH)_3_Br and LiOH, are indicated with colored lines. The stoichiometric ranges of these compounds are not represented by the widths of the lines, but are expected to be narrow. Li_4_(OH)_3_Br is shown as a peritectic compound, forming from liquid + LiOH at ∼290 °C. Horizontal lines at 280 and 230 °C (75 < *X* mol % LiOH < 100) have been replicated from the original phase diagram, and were plotted in response to observed DSC peaks. (b) Crystal structure of Li_4_(OH)_3_Br proposed by Hönnerscheid,^17^ in which _∞_^2^ [Li_4_(OH)_3_^+^] layers are separated by bromine anions.

Li_4_(OH)_3_Br is a compound found in the LiBr-LiOH phase diagram with a melting point around 290 °C.^8,15−18^ Although various LiBr-LiOH phase diagrams have been proposed, there is agreement that Li_4_(OH)_3_Br is a peritectic compound forming from liquid + LiOH.^8,18^ The most recent iteration is shown in Figure 1a. The crystal structure of Li_4_(OH)_3_Br, determined by Hönnerscheid et al., belongs to the *P*2_1_/*m* space group and is shown in Figure 1b.^17^

Despite the promising calculations, experimental melting enthalpies above 250 J g^–1^ have not been obtained to date.^8,10−12^ A possible factor contributing to this is the slow kinetics of peritectic reactions, often leading to incomplete formation of the peritectic phase. Mahroug et al. used X-ray diffraction (XRD) to argue that this is not the case, and that complete reaction occurs in samples cooled at rates between 0.5 to 50 K min^–1^. This suggests that a direct transformation in which the peritectic phase directly nucleates in the liquid upon cooling occurs instead, as has been reported in various binary systems.^19−23^ However, it should be noted that they chose to disregard transmission electron microscopy (TEM) and energy dispersive X-ray spectroscopy (EDS) images which showed inhomogeneities in composition, indicating that there could be multiple phases present.^12^ Legros et al. proposed a polymorph may sometimes form depending on the synthesis precursors used, and suggested that the presence of this phase may explain the lower storage capacities observed experimentally.^11^

In spite of the subtheoretical values obtained experimentally, Li_4_(OH)_3_Br still demonstrates excellent melting enthalpies for PCMs in the range 280 to 310 °C.^2−4^ When compared to NaNO_3_, the most highly researched option of TES for DSG CSP, Li_4_(OH)_3_Br demonstrates improved energy density and reduced volume changes, which could potentially offer infrastructure savings and improved lifetimes.^12,24^ It is apparent that, despite showing promising thermal storage behavior, a complete understanding of Li_4_(OH)_3_Br is missing.

In this paper, we conduct an in-depth study of Li_4_(OH)_3_Br. Supported by theoretical modeling, a combination of heat treatments, variable-temperature XRD and differential scanning calorimetry (DSC) allows us to build on the current understanding of Li_4_(OH)_3_Br and the LiBr-LiOH system. We demonstrate that previous reports of Li_4_(OH)_3_Br correspond to a hydrated compound, and address the implications of this on thermal energy storage. We find that Li_4_(OH)_3_Br cannot easily be obtained phase-pure from direct cooling procedures as a result of nonequilibrium solidification. We design a route to synthesize the phase with high purity, enabling subsequent characterization of the structure and thermal behavior of Li_4_(OH)_3_Br, supported by theoretical modeling.

## Synthesis

**Figure 2:**
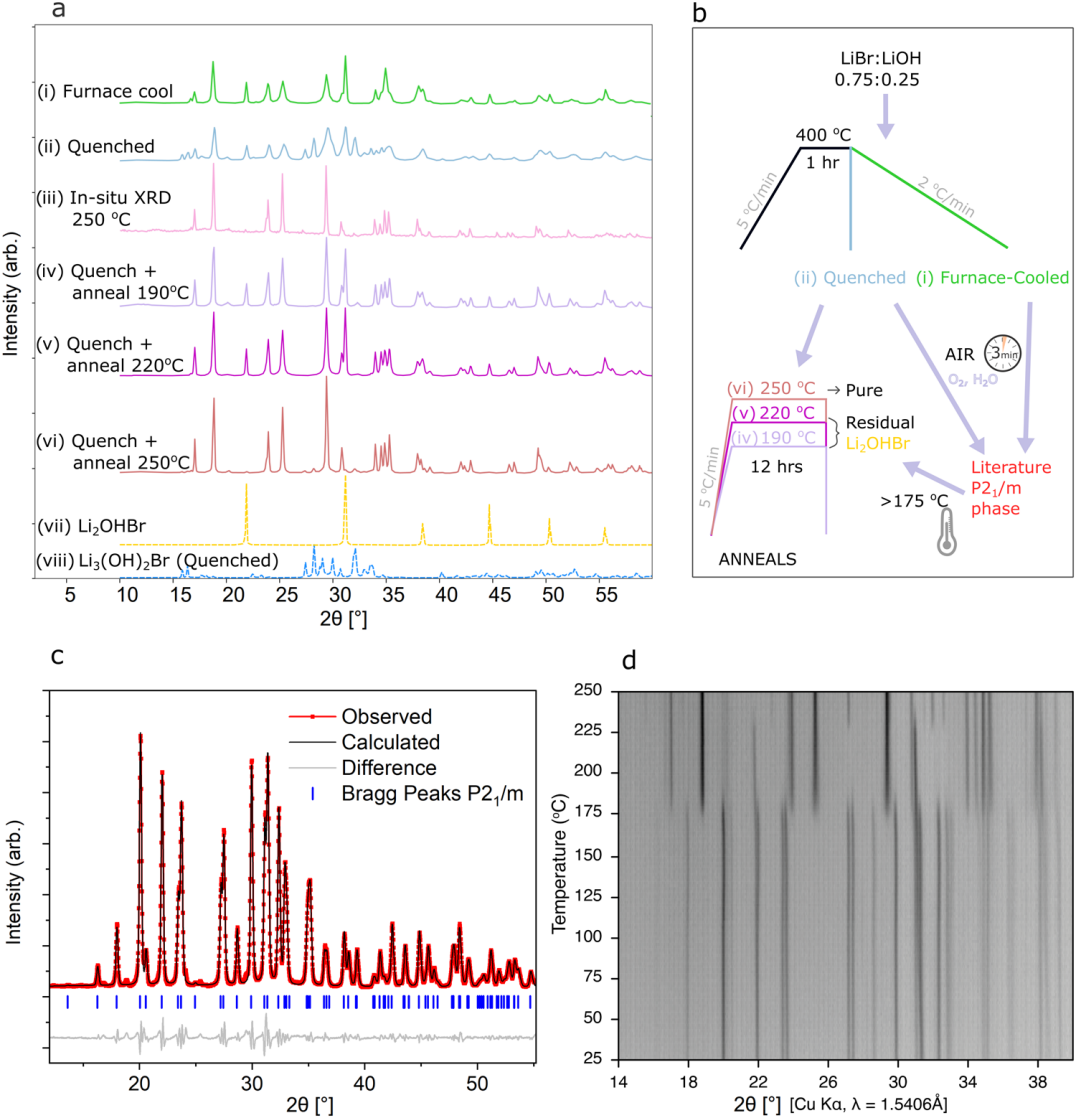
Synthesis Investigations. (a) X-ray diffraction patterns obtained for Li_4_(OH)_3_Br synthesized under a variety of conditions. The synthesis conditions are found to have a big impact on the phase-purity of the obtained samples. For ease of comparison, the diffraction patterns of Li_2_OHBr and metastable quenched Li_3_(OH)_2_Br are included to highlight conditions under which residual impurities remain. (b) Schematic showing the relationship between the different synthesis conditions presented in part a and described in text. (c) Powder XRD pattern of air-exposed Li_4_(OH)_3_Br and corresponding Pawley refinement (*R*_wp_ = 7.0%) to the *P*2_1_/*m* crystal structure reported in literature, with a difference plot included beneath.^17^ The Bragg peak positions corresponding to the *P*2_1_/*m* phase are indicated with ticks. (d) Variable-temperature XRD patterns of air-exposed *P*2_1_/*m* Li_4_(OH)_3_Br heated from room temperature to 250 °C, displayed on a film plot. Reformation of the nonair-exposed diffraction pattern occurs at ∼175 °C. The disappearance and appearance of Li_2_OHBr and Li_3_(OH)_2_Br respectively can be seen upon further heating at ∼230 °C.

To produce Li_4_(OH)_3_Br, a synthesis route representative of those in literature was carried out. Stoichiometric ratios of anhydrous LiBr and LiOH were ground together, heated to 400 °C for 1 h with a ramp rate of 5 °C min^–1^, furnace-cooled at 2 °C min^–1^ inside of a glovebox, and ground into a powder using an agate mortar and pestle. An XRD pattern obtained from these samples, also taken under glovebox atmosphere, is shown in Figure 2ai. A schematic of the process is shown in Figure 2b. The XRD pattern does not match previous literature reports of *P*2_1_/*m* Li_4_(OH)_3_Br,^8,10−12,17^ however it has previously been found by Legros et al., who speculated that it might be a polymorph of Li_4_(OH)_3_Br.^11^ We believe that the pattern observed actually corresponds to a mixture of phases, containing a large fraction of the Li_2_OHBr antiperovskite phase, shown for comparison in Figure 2avii, and the true thermodynamically stable Li_4_(OH)_3_Br phase. The solidification of Li_4_(OH)_3_Br is proposed to be peritectic through the reaction: liquid + LiOH →Li_4_(OH)_3_Br.^8,18^ Given the unfavorable kinetics of peritectic reactions, it can be challenging to achieve equilibrium conditions, and so the presence of nonequilibrium phases such as Li_2_OHBr in samples at room-temperature is not surprising. The implications of nonequilibrium solidification are an important consideration in the use of peritectic compounds for LHS, as the enthalpy of melting less energetically stable phases differs to that of their equilibrium counterparts.

The samples were found to be very air sensitive. A furnace-cooled sample was exposed to air for 3 min, before returning to the glovebox and recording XRD patterns under inert atmosphere. The diffraction pattern displayed a different set of peaks, which correspond to the *P*2_1_/*m* crystal structure commonly reported in literature. Pawley refinements for the *P*2_1_/*m* structure, shown in Figure 2c (Table S1), gave a unit cell of *a* = 5.4594(3) Å, *b* = 7.5922(3) Å, *c* = 6.5090(3) Å, β = 93.841(2) °, in excellent agreement with literature.^17^ As such, we believe that the previously reported phase corresponds to a hydrated state and not the true Li_4_(OH)_3_Br phase. When quenching Li_4_(OH)_3_Br samples to room temperature from the liquid state at 400 °C, a different diffraction pattern was found, as shown in Figure 2aii. Nevertheless, the same *P*2_1_/*m* peaks were observed to form from quenched samples upon air exposure (Figure S1), further supporting that the phase reported in literature is hydrated. It is likely that XRD setups employed in previous literature were not sufficiently airtight to avoid hydration, explaining why Mahroug et al. believed they had obtained pure Li_4_(OH)_3_Br regardless of cooling rate.^12^

To understand what happens to the *P*2_1_/*m* phase upon heating, variable-temperature (VT) XRD was conducted on a pellet of pressed Li_4_(OH)_3_Br powder with the air-exposed *P*2_1_/*m* phase, heated at 1 °C min^–1^ under argon flow. As shown in the film plot in Figure 2d, the *P*2_1_/*m* diffraction peaks disappear between 170 and 190 °C. Significantly, this means that the impressive enthalpies of melting predicted from calculations based on this phase, do not apply. The diffraction pattern which forms at 170 °C resembles the anhydrous furnace-cooled phase. At ∼230 °C, some peaks disappear and new peaks appear, corresponding to the antiperovskite Li_2_OHBr and high-temperature Li_3_(OH)_2_Br phases, respectively.^25^ Diffraction patterns extracted at 200 and 240 °C illustrate this clearly in Figure S2. During an *in situ* anneal for 1 h at 250 °C, the intensity of the Li_3_(OH)_2_Br peaks decreased, leaving the diffraction pattern shown in Figure 2aiii. These same peaks are observed above ∼220 °C in synchrotron VT-XRD of unexposed (anhydrous) samples quenched from the melt and reheated *in situ* (Figure S3). Hence, it can be implied that these remaining peaks correspond to the true Li_4_(OH)_3_Br phase. Although there appears to be some reversibility of the hydration process upon heating, it is worth noting that the hydrated *P*2_1_/*m* phase was not stable in air, and decomposed into new products upon continued air exposure (Figure S1).

To see whether phase-pure samples of anhydrous Li_4_(OH)_3_Br could be obtained directly from the melt through an alternative route, samples were quenched to room-temperature by removing from the furnace at 400 °C. By providing a large undercooling, it was hoped that direct nucleation of Li_4_(OH)_3_Br would take place, rather than formation through peritectic reaction or peritectic transformation. This has been previously reported for other peritectic materials.^19−23^ As has already been mentioned, the resulting diffraction pattern (Figure 2aii) does not match that of the furnace-cooled sample, or of phase-pure Li_4_(OH)_3_Br. Further investigation (discussed in Supporting Information Note 1, Figure S4) revealed that quenched samples do not represent the equilibrium room-temperature state, and, to the best of our knowledge, there is only one Li_4_(OH)_3_Br phase between 250 °C and room temperature.

Since pure room-temperature Li_4_(OH)_3_Br could not be produced directly from the melt, a series of anneals and quenches were carried out on different samples quenched from the liquid state. The corresponding XRD patterns for these samples are shown in Figures 2aiv–vi. Quenched samples have fine microstructures, maximizing the contact area between reacting phases and reducing diffusion distances, and so were chosen for the starting material. Approximately 0.5 g of samples were ground into a powder using a mortar and pestle, annealed for 12 h at the stated temperature, and then quenched back to room-temperature to try and ‘freeze-in’ the phase formed during the anneal, as shown schematically in Figure 2b. In anneals below 250 °C, a large amount of antiperovskite Li_2_OHBr was found in samples, likely due to insufficient kinetics to obtain equilibrium at these temperatures.

Annealing at 250 °C produced Li_4_(OH)_3_Br. Minor impurity peaks resembling the diffraction pattern observed for metastable quenched Li_3_(OH)_2_Br^25^ (Figure 2aviii) were found to form, irrespective of the cooling rate employed following the anneal. Employing the same quench and anneal procedure on 80 mol % LiOH composition yields samples containing a large fraction of LiOH, in addition to the same impurity peaks and Li_4_(OH)_3_Br, indicating that the composition cannot be compensated in this way (Figure S5). It is possible that the phase’s stoichiometry does not lie exactly on the binary phase diagram, perhaps explaining the difficulty in making completely phase-pure samples from LiBr and LiOH precursors through this approach. Nevertheless, only a minor fraction of impurity exists in Li_4_(OH)_3_Br produced from the annealed 75 mol % LiOH samples, supported further by Raman spectroscopy measurements (Figure S6).

The complex relationship between processing route (Figure 2b) and phase-purity (Figure 2a) found in this work highlights the importance of Li_4_(OH)_3_Br synthesis conditions. Implications of these findings on the current understanding of the LiBr-LiOH phase diagram are considered in Supporting Information Note 2. Previous reports have not synthesized pure Li_4_(OH)_3_Br, and the properties have yet to be established. Electrochemical impedance spectroscopy (EIS) revealed Li_4_(OH)_3_Br to be ionically resistive, unlike previously suggested^16^ (see Supporting Information Note 3).

## Crystal Structure

To determine the crystal structure of the Li_4_(OH)_3_Br phase, XRD peak indexing and space group searching was conducted using TOPAS-ACADEMIC software.^26^ Peaks corresponding to the residual Li_3_(OH)_2_Br were excluded from analysis. The diffraction pattern at 250 °C could be accounted for by an orthorhombic unit cell ∼10.4 Å × 5.3 Å × 5.3 Å. Systematic absences were consistent with the *Pmnm* space group symmetry. A Pawley refinement carried out using this space group setting gave an excellent fit to the data and a refined unit cell metric of *a* = 10.4099(3) Å, *b* = 5.30384(15) Å, *c* = 5.27776(13) Å (Table S2).

Independently, ab initio random structure searching (AIRSS) was employed to find low-energy structures for Li_4_(OH)_3_Br, and optimized using a CHGNet machine-learned force field fine-tuned for the Li–O–H-Br phase space. A possible layered crystal structure aligning well with the experimentally determined unit cell was consistently identified, shown in Figure S8 and detailed in Table S3. The agreement between the data-driven and physics-based approaches support the robustness of these findings.

**Figure 3:**
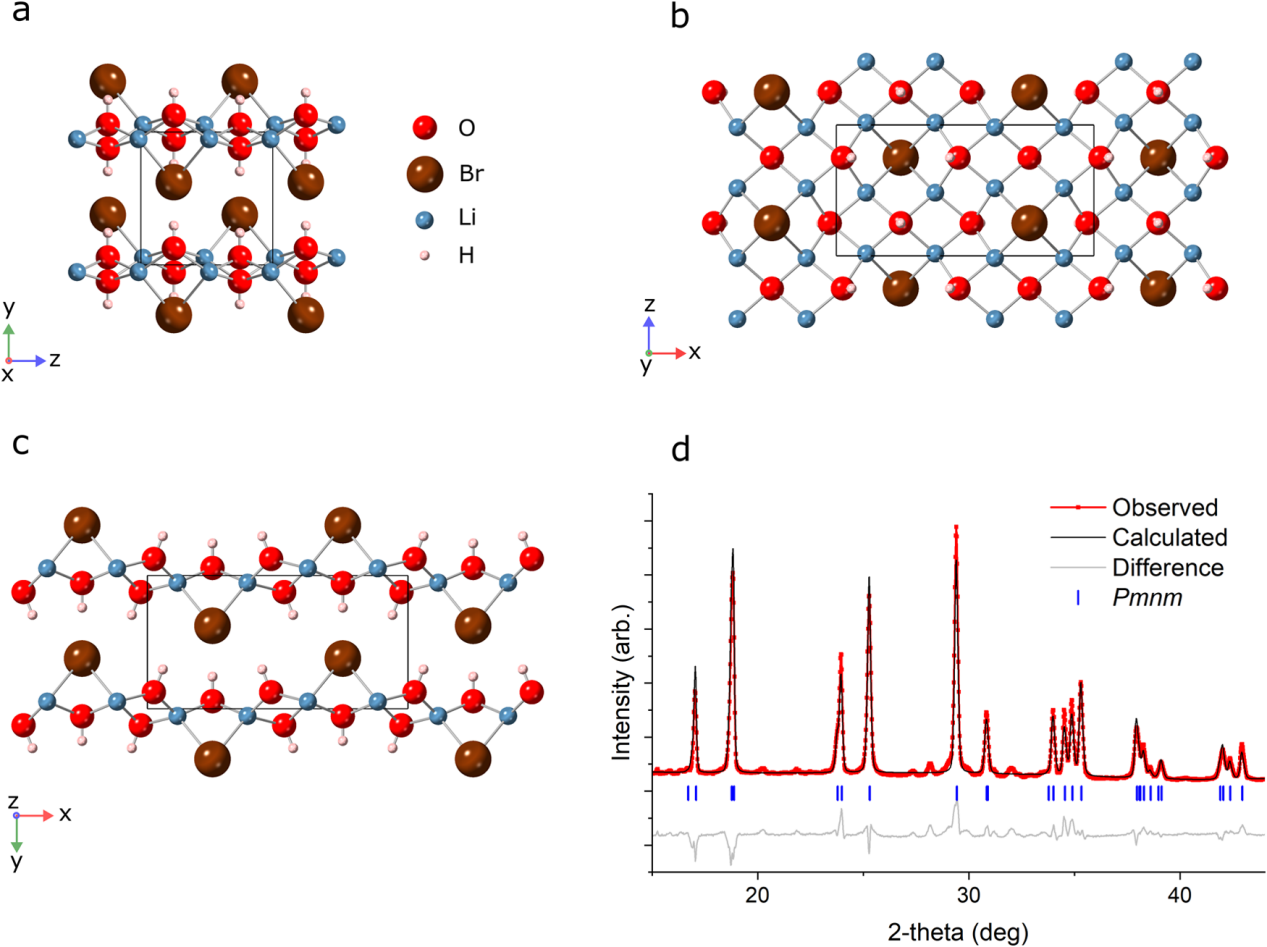
Crystal Structure. *Pmnm* structural model for Li_4_(OH)_3_Br viewed along (a) [100], (b) [010] and (c) [001]. The unit cell is indicated with a black box superimposed on the crystal structure. (d) Powder XRD pattern of room-temperature Li_4_(OH)_3_Br produced through the 250 °C annealing route and corresponding Rietveld refinement to the predicted crystal structure. The resulting difference plot is provided beneath the data. A *R*_wp_ of 11.6% is obtained using the crystallographic information provided in Table 1 for the refinement. Bragg peaks arising from the *Pmnm* crystal structure are indicated with ticks. The unrefined minority peaks likely correspond to metastable quenched Li_3_(OH)_2_Br.

A constrained Rietveld refinement was then implemented in which the atom coordinates of the AIRSS solution were combined with the Pawley cell metric to give a structural model for Li_4_(OH)_3_Br, shown in Figure 3a–c. Subsequent refinement of this model involved allowing variation in cell metric, peak-shape parameters, background parameters and the positional coordinates of Br. The positions of Li, O and H within the unit cell were not refined as a consequence of their low X-ray scattering cross sections. The Rietveld refinement corresponding to the 250 °C annealed room-temperature sample is shown in Figure 3d and the crystallographic parameters detailed in Table 1. A refinement for the *in situ* measurements at 250 °C is provided in Figure S3 and Tables S4 and S5. An increase in lattice parameter was seen between the room-temperature and *in situ* 250 °C samples, particularly along the *a*-axis (Table 2). To clarify further information on the crystal structure, complementary techniques such as neutron diffraction should be employed in future work.

**Table 1:** Crystallographic Parameters for the *Pmnm* Structure of Li_4_(OH)_3_Br at Room Temperature Produced via the 250 °C Anneal Route, from the Fit Shown in Figure 3d^a^

*a* (Å)	10.3761(11)				
*b* (Å)	5.3028(5)				
*c* (Å)	5.2681(5)				
*V* (Å^3^)	289.86(5)				
atom	Wyckoff position	*x*	*y*	*z*	occupancy
Br1	2b	0.25	0.3727(8)	0.75	1
O1	4f	–0.02508	0.12415	0.25	1
O2	2a	0.25	–0.05675	0.25	1
Li1	8g	0.61587	–0.053665	0.5171	1
H1	4f	–0.05934	0.2956	0.25	1
H2	2a	0.25	0.75881	0.25	1

a Refined parameters are indicated with errors in brackets.

**Table 2:** Lattice Parameters Determined from Rietveld Refinements of Li_4_(OH)_3_Br at Room Temperature and 250 °C

	room temperature	250 °C (*in situ*)
*a* (Å)	10.3761(11)	10.41106(17)
*b* (Å)	5.3028(5)	5.30442(7)
*c* (Å)	5.2681(5)	5.27854(7)

## Latent Heat

**Figure 4:**
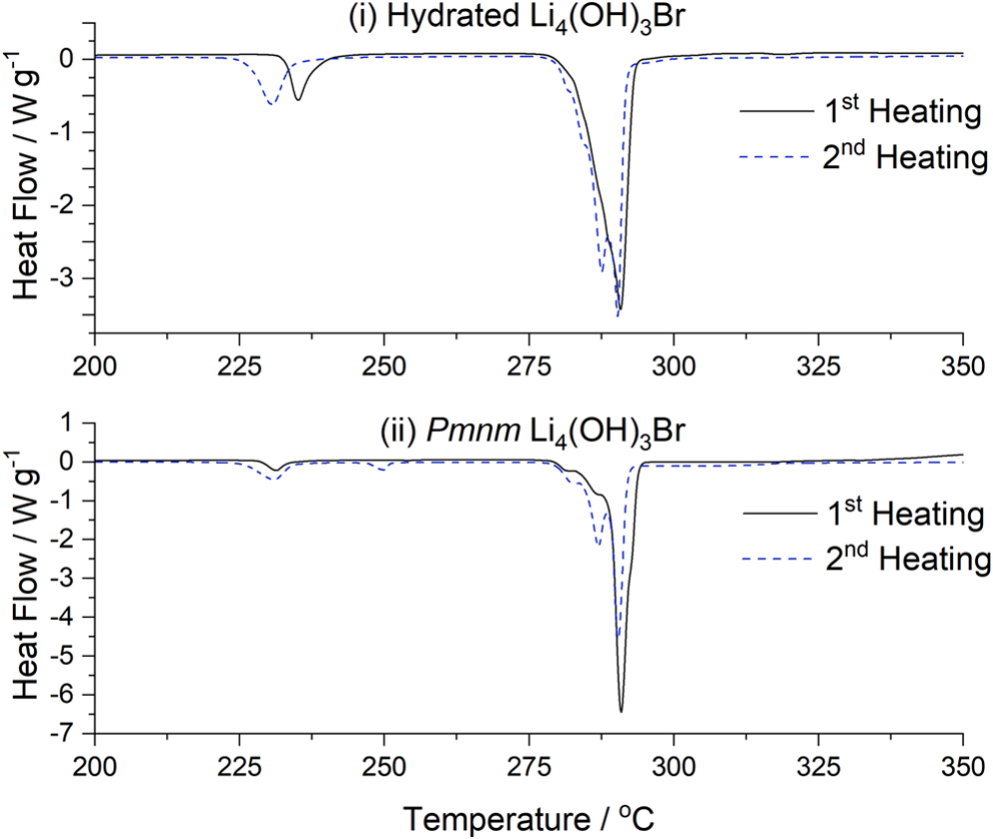
Latent Heat Storage. DSC heating profiles of (i) hydrated *P*2_1_/*m* Li_4_(OH)_3_Br, and (ii) *Pmnm* Li_4_(OH)_3_Br, showing various endothermic processes occurring between 200 and 350 °C. Two heating cycles are shown in each case. The second heating cycle of the *Pmnm* Li_4_(OH)_3_Br varies from the first due to the nonequilibrium solidification which occurs after the first heating cycle.

DSC was employed to study the thermal behavior of Li_4_(OH)_3_Br. The 75 mol % LiOH samples annealed at 250 °C followed by quenching were chosen as the purest samples. For comparison, samples exhibiting the hydrated *P*2_1_/*m* phase were also investigated. In each instance, a small amount of powder (<2.5 mg) was hermetically sealed in an aluminum pan in an argon glovebox, and samples were measured heating and cooling between 25 and 400 °C at 5 °C min^–1^. Measurements are shown in Figure 4. The DSC behavior for the hydrated samples aligned with that seen in the VT-XRD: a small endothermic peak corresponding to dehydration was observed at ∼ 175 °C (Figure S9), resulting in samples containing a mixture of Li_4_(OH)_3_Br and Li_2_OHBr. At ∼230 °C, the Li_2_OHBr converts to Li_3_(OH)_2_Br, indicated by another endothermic peak. The signal around 280 °C appears to be a superposition of peaks, including decomposition of the remaining Li_3_(OH)_2_Br, and melting of the Li_4_(OH)_3_Br. Experimentally, Li_3_(OH)_2_Br has been shown to bypass the formation of Li_4_(OH)_3_Br indicated on the phase diagram above 280 °C, instead melting directly.^25^

In DSC of the *Pmnm* Li_4_(OH)_3_Br, the peaks corresponding to the formation and decomposition of Li_3_(OH)_2_Br only appear very weakly. If fully pure Li_4_(OH)_3_Br, these peaks would not be expected to occur at all. Instead, the minor impurity phase observed likely transforms to *P*6_3_/*mmc* Li_3_OH_2_Br between ∼230 and ∼280 °C, giving rise to the small peaks seen.

The enthalpies associated with the combined collection of peaks between 275 and 295 °C are reported for each heating run in Table 3, consistent with the approach taken in literature. We find an enthalpy of 263 ± 3 J g^–1^ associated with the melting of the anhydrous *Pmnm* samples, surpassing values reported previously (≤250 J g^–1^).^8,10,12^ Incorrect phases were used in the previous calculations predicting a melting enthalpy of 804 J g^–1^, while experimentally measured enthalpies are much lower. The theoretical melting enthalpy for the crystal structure proposed here-within was estimated to be 260 J g^–1^ (Figure S10), which is in excellent agreement with our experimental findings. Once the *Pmnm* Li_4_(OH)_3_Br has been melted and is cooled at 5 °C min^–1^ in the DSC, the sample undergoes nonequilibrium solidification, and the subsequent heating cycle gives rise to much stronger peaks associated with the nonequilibrium processes. Consequently, the measured enthalpy change decreases considerably, as the fraction of material undergoing the high-enthalpy Li_4_(OH)_3_Br → Liquid + LiOH transition decreases. This contrast between the first and second heating cycle is apparent in the difference in enthalpy values reported in Table 3. Although this suggests higher enthalpies than previously reported may be obtainable, this is not practically relevant without an approach to obtain equilibrium single-phase Li_4_(OH)_3_Br in-use.

**Table 3:** Phase Transition Enthalpies for the Li_4_(OH)_3_Br Samples shown in Figure 4. Errors of 1% are Expected for the Reported Enthalpy Values

		enthalpy of phase transition/J g^–1^	
	sample	230 °C	275–295 °C
hydrated	first heating	31	231
	second heating	39	229
*Pmnm*	first heating	12	263
	second heating	29	200

## Conclusions

In this paper, we conduct an in-depth study of Li_4_(OH)_3_Br. We find that the crystal structure for Li_4_(OH)_3_Br established in literature in fact corresponds to a metastable hydrated state, which ceases to exist upon heating to ∼175 °C. Samples of the thermodynamically stable Li_4_(OH)_3_Br phase are synthesized via annealing and investigated with respect to their application in TES. An orthorhombic crystal structure with lattice parameters of *a* = 10.38 Å, *b* = 5.30 Å, *c* = 5.27 Å and the *Pmnm* space group is found through diffraction studies, and a potential crystal structure is suggested with the help of theoretical modeling. These findings indicate that previous calculations for the melting enthalpy of Li_4_(OH)_3_Br are not applicable. An experimental melting enthalpy of 263 ± 3 J g^–1^ is found for Li_4_(OH)_3_Br, in good agreement with calculations for the proposed Li_4_(OH)_3_Br crystal structure reported herein. Discrepancies with previous reports for the melting enthalpy of Li_4_(OH)_3_Br (≤250 °C J g^–1^) arise due to the tendency of peritectic compounds to undergo nonequilibrium solidification, exemplified by the presence of other phases in samples prior to anneals. Although the experimentally observed latent heats still represent substantial energy storage capacities, phase segregation could pose challenges to the long-term cycling of Li_4_(OH)_3_Br. This issue may need to be addressed through mitigating strategies such as periodic annealing, stirring or encapsulation.

If Li_4_(OH)_3_Br is to be considered further as a candidate PCM for TES, future work should focus on better understanding the high-temperature portion of the phase diagram, such that solidification can be understood better and potentially controlled. Approaches to promote equilibrium solidification, perhaps through the use of nucleating agents or optimized cooling conditions, should be explored, and further characterization of the Li_4_(OH)_3_Br phase, such as its thermal conductivity, would be valuable.

## Supporting Information Available

Experimental and computational details, additional results including XRD, Raman spectroscopy and DSC, and further discussion referenced in the main text (PDF)
