# Supplementary material for: Synthesis and Characterization of Li4(OH)3Br for Thermal Energy Storage
Source: ACS Appl Energy Mater. 2025 Apr 4;8(8):5353–9. doi: 10.1021/acsaem.5c00359 (PMC12042160; doi:10.1021/acsaem.5c00359)
Supplement: Supplementary file 1 — ae5c00359_si_001.pdf [file ae5c00359_si_001.pdf]

Supporting Information - Synthesis and characterization of $Li_4(OH)_3Br$ for thermal energy storage

Emily Milan[1], James A. Quirk[2], John Cattermull[1,3], Andrew L. Goodwin[3], James A. Dawson[2], and Mauro Pasta*[1]

[1]Department of Materials, University of Oxford, Oxford, OX1 3PH, UK

[2]Chemistry – School of Natural and Environmental Sciences, Newcastle University, Newcastle upon Tyne, NE1 7RU, UK

[3]Department of Chemistry, University of Oxford, Oxford, OX1 3QR, United Kingdom

*Corresponding author: mauro.pasta@materials.ox.ac.uk

Experimental Details

Synthesis

Anhydrous LiOH (98%, Sigma-Aldrich) and LiBr ($\geq$ 99%, Sigma-Aldrich) were weighed out in the correct molar ratio for the stoichiometry being synthesized, accounting for the purity of the precursors. The weighed-out samples were then ground using an agate mortar and pestle for ∼5 minutes to ensure a homogeneous mixture and short diffusion distances. The resulting mixture was placed into alumina crucibles and heated in a muffle furnace to the molten state at 400 °C for 1 hour, with a ramp rate of 5 °C/min. "Furnace-cooled" samples were cooled back to room temperature at 2 °C/min, whereas "quenched" samples were removed from the furnace and placed on the stainless steel floor of the glovebox (∼ 25 °C) to cool naturally to room temperature. After cooling, the samples were ground into a powder using an agate mortar and pestle. For anneals, quenched samples were crushed into a powder and reheated to the desired temperature with a ramp rate of 5 °C/min.

Unless stated otherwise, all work was carried out under inert conditions due to the highly air-sensitive nature of the investigated compounds. Samples were handled in argon-filled gloveboxes (MBraun, H_2O < 0.5ppm, O_2 < 0.5ppm) and were transferred between gloveboxes in closed glass vials, sealed with parafilm. A vacuum oven (∼1mbar, 70 ^oC) was used to dry utensils and consumables for a minimum of 4 hours prior to use.

XRD

Most measurements were taken on a Rigaku Miniflex diffractometer (Cu Kα) in a nitrogen-filled glovebox (MBraun, H_2O < 0.5ppm, O_2 < 0.5ppm) on powder samples loaded on a single crystal silicon holder to minimize non-crystalline background contributions.

The in-situ air-exposure XRD measurements in Figure S1 were made using a Rigaku Smartlab diffractometer (Cu Kα) on powder samples loaded in a borosilicate capillary.

The variable-temperature XRD measurements in Figure 2c were also made using the Rigaku Smartlab diffractometer (Cu Kα) on powder pressed into a 10 mm pellet and loaded onto a heating stage under argon flow. Samples were heated and cooled at a rate of 1 K/min.

Synchrotron powder XRD measurements were carried out on the I11 beamline at Diamond Light Source (λ = 0.82311 Å). Powders were sealed in 0.5 mm borosilicate glass capillaries (wall thickness 0.01 mm), ensuring protection from air exposure. An FMB Oxford cyberstar hot air blower was used to

apply a continuous heating rate of 6 K/min during which measurements were taken at approximately 2.5 °C intervals. Diffraction patterns were collected in capillary transmission geometry using the Mythen2 Position Sensitive Detector, two data collections of 5 seconds each were taken at angles 0.25 degrees apart, then summed to account for gaps in the detector coverage.

Pawley and Rietveld refinements were carried out using TOPAS-Academic software [1]. Unit cell, background and peak shape parameters were allowed to refine freely. In Rietveld refinements, the atomic positions in $Li_4(OH)_3Br$ were fixed for the lighter atoms, O, Li and H. Where $Li_3(OH)_2Br$ is involved, the atomic positions were taken from the structural model for $Li_3(OH)_2Br$ reported in reference [2]. At 250 °C, a thermal displacement parameter was introduced. In order to reduce the number of free parameters, the thermal parameter was constrained to be equal across elements.

DSC

DSC measurements were taken on a TA Instruments DSC25, using ramp rates of 5 °C/min. Samples were hermetically-sealed in aluminum crucibles in argon gloveboxes to ensure full protection from air exposure. Peak integration for enthalpy determination was carried out using OriginLab software.

EIS

For electrochemical measurements, powders were cold-pressed into 5mm diameter pellets for 3 minutes at 370 MPa. Nickel foil (Advent Materials, 99.95 %, 0.0125 mm) blocking electrodes were placed on either side of the electrolyte pellet. A custom-built cell applies a uniaxial pressure of 70 MPa via stainless steel pistons on each side of the cell, to ensure good contact.

For potentiostatic electrochemical impedance spectroscopy (PEIS), cells were connected to a BioLogic MTZ35 frequency response analyzer in a two-point probe configuration. Measurements were taken in a frequency range of 35 MHz to 0.1 Hz with a voltage amplitude of 10 mV. To control the temperature, the cell was heated in a muffle furnace and allowed to dwell for 45 minutes at each temperature prior to measurement.

Raman Spectroscopy

A Renishaw inVia Reflex laser confocal Raman microscope with a 532 nm laser and 1800 lines mm^{-1} grating was used to measure samples by focusing the laser through a glass vial onto powder contained within. Spectra were recorded for 20 accumulations with a laser power of 150 mW and a 1 s exposure

time, followed by background removal using the Renishaw WiRE 5.5 software.

Computational Details

A preliminary ab-initio random structure searching (AIRSS)[3] run was performed on several hundred geometries for $Li_3(OH)_2Br$ and $Li_4(OH)_3Br$ using the pre-trained CHGNet foundation model. Then, a CHGNet model was fine-tuned for Li-O-H-Br systems by training against a set of molecular dynamics trajectories. The systems were: solid Li_2OHBr, $Li_3(OH)_2Br$, and $Li_4(OH)_3Br$ at 900 K with NPT ensemble; and molten Li_2OHBr, $Li_3(OH)_2Br$, and $Li_4(OH)_3Br$ at 2000 K with NVT ensemble. The fine-tuned model was then used for the final AIRSS to determine the stable structure in this work and all further MD runs. Due to the importance of H-bonding in materials containing OH species, dispersion was treated using the DFT-D3 method [4] using the implementation in `torch-dftd` [5]. Analysis of the MD trajectories was performed with `Pymatgen` [6] and the Atomic Simulation Environment [7].

All geometry optimizations were carried out until forces on ions were less than 0.05 eV/Å. The time-step for molecular dynamics was 0.5 fs to ensure numerical stability when integrating equations of motion due to the small mass and rapid acceleration of protons. All MD calculations used a supercell that was a $2 \times 4 \times 4$ expansion of the unit cell. Enthalpy of melting is determined using the same procedure as Alvarez et. al [8]. The structure was melted and equilibrated at 1200 K for a total of 50 ps. For temperatures between 230 °C and 260 °C in increments of 10 °C, MD was performed on the solid material and the liquid material, for at least 100 ps with 25 ps of equilibration to allow the material to come to the correct temperature. The mean enthalpy across each run was taken then, by fitting straight lines to the solid and the liquid branch, the enthalpy of melting can be determined from the difference between the two lines.

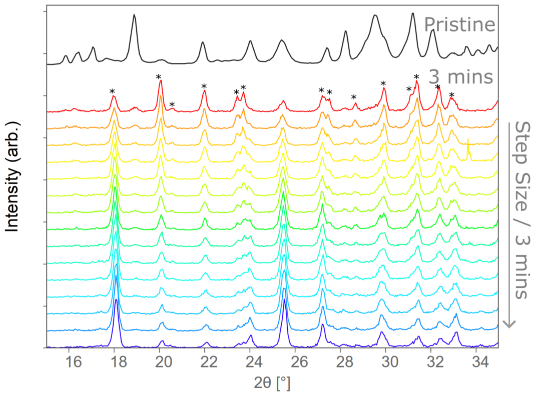

Figure S1: Diffraction patterns demonstrating the instability of quenched $Li_4(OH)_3Br$ in air. The top 'pristine' pattern shows a diffraction pattern taken under inert conditions. Subsequent patterns correspond to continuous XRD scans with a duration of 3 minutes taken on samples in air. A large reduction in the starting phase, and the formation of peaks corresponding to the $P2_1/m$ phase reported in literature (labeled with asterisks) are observed in as little as 3 minutes exposure

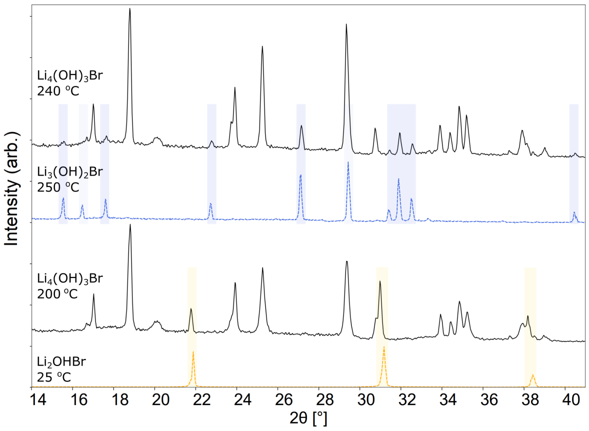

Figure S2: XRD patterns of $Li_4(OH)_3Br$ at 200 °C and 240 °C, extracted from the film plot in Figure 2d in the main text, along with diffraction patterns for $Li_3(OH)_2Br$ and Li_2OHBr references. Li_2OHBr impurity is present at 200 °C which is replaced by $Li_3(OH)_2Br$ in the diffraction pattern at 240 °C. Note that there is a slight peak shift between pure Li_2OHBr and the Li_2OHBr impurity in the $Li_4(OH)_3Br$ due to the different measurement temperatures.

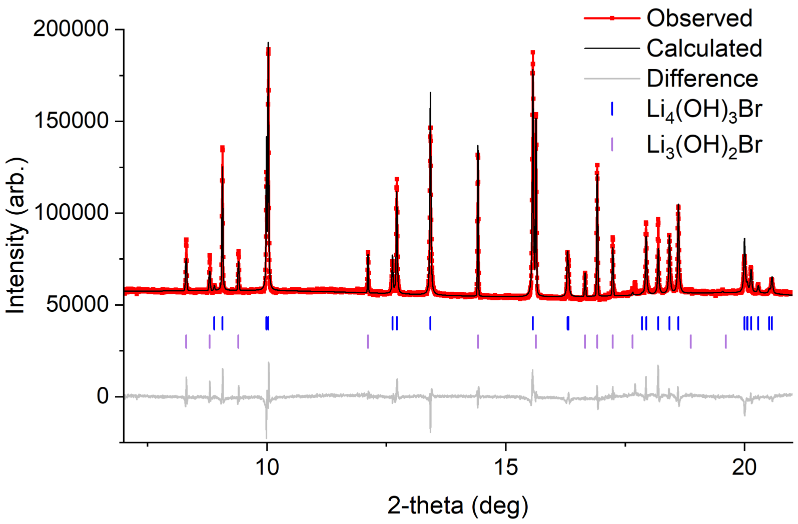

Figure S3: Synchrotron powder XRD pattern of a 75 mol % LiOH sample at 250 °C and corresponding Rietveld refinement (R_{wp} = 1.5 %) for *Pmnm* $Li_4(OH)_3Br$ and $P6_3/mmc$ $Li_3(OH)_2Br$, the Bragg peak positions of which are indicated with ticks. The resulting difference curve is offset beneath the data. Samples were quenched to room temperature from 400 °C and reheated at 6 K/min. The sample contains a large fraction of $P6_3/mmc$ $Li_3(OH)_2Br$ [2] due to the high heating rate employed meaning equilibrium conditions are not attained. The $Li_4(OH)_3Br$ was fitted using the crystal structure model proposed in Figure 3, and the refinement details are provided in Tables S4 and S5.

Supporting Information Note 1 - Quenched $Li_4(OH)_3Br$

75 mol % LiOH sample was melted at 400 °C and quenched to room temperature to establish whether the equilibrium room-temperature phase could be directly nucleated, rather than a slower solidification route passing through other phase fields which may kinetically hinder formation of the room-temperature phase. The diffraction pattern obtained contains additional peaks to the pure $Li_4(OH)_3Br$ phase identified in Figure 2aiii. This could indicate the presence of a low temperature phase field containing another $Li_4(OH)_3Br$ polymorph, for example, as is suggested by Mahroug's phase diagram below 230 °C.

Upon heating quenched samples, a series of changes occur from $\sim$ 80 °C, observed in VT-XRD and DSC measurements shown in Figure S4a and b. As such, the quenched state is not stable to 230 °C as the polymorph indicated on the phase diagram would be. The large number of independently-occurring changes imply the presence of several phases and likely correspond to a series of metastable steps.

From ex-situ XRD and Raman spectroscopy comparisons, shown in Figures S4c and d, it is possible to see that the quenched samples correspond to a mixture of phases containing Li_2OHBr, LiOH and metastable quenched $Li_3(OH)_2Br$, as well as the previously observed $Li_4(OH)_3Br$ phase. This indicates that direct nucleation of the $Li_4(OH)_3Br$ phase does not occur, as it had been hoped would. Instead, some $Li_3(OH)_2Br$ appears to nucleate at high temperatures, and the high cooling rate means that the kinetics are not sufficiently fast for transformation to $Li_4(OH)_3Br$ upon cooling. $Li_3(OH)_2Br$ is a high-temperature phase which has been demonstrated to decompose into a metastable state upon quenching to room temperature, as is observed here [2].

As such, we believe that $Li_4(OH)_3Br$ samples obtained from quenching do not represent equilibrium conditions at room temperature, and to the best of our knowledge, there is only one $Li_4(OH)_3Br$ phase between 250 °C and room temperature.

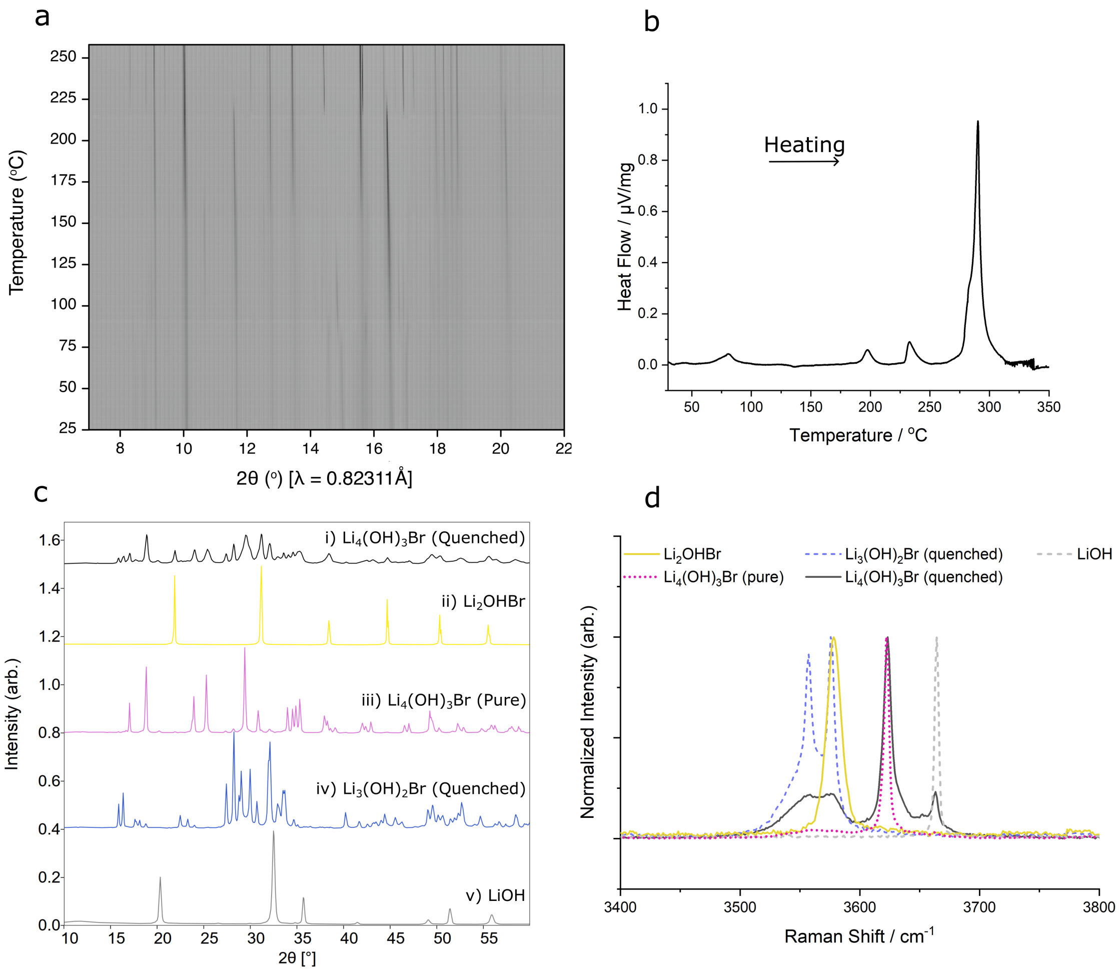

Figure S4: Quenched $Li_4(OH)_3Br$. a) Synchrotron variable-temperature XRD patterns ($\lambda = 0.82311$ Å) of quenched $Li_4(OH)_3Br$ showing a series of changes upon heating at 6 K/min. b) Heating DSC of quenched $Li_4(OH)_3Br$ at a rate of 5 K/min demonstrating a series of small endothermic peaks prior to melting at 300 °C. c) XRD measurements comparing quenched $Li_4(OH)_3Br$ with the phase-pure compounds Li_2OHBr, $Li_4(OH)_3Br$, LiOH and metastable quenched $Li_3(OH)_2Br$. The quenched $Li_4(OH)_3Br$ consists of a combination of these phases. d) Raman spectroscopy measurements comparing the same compounds as in c.

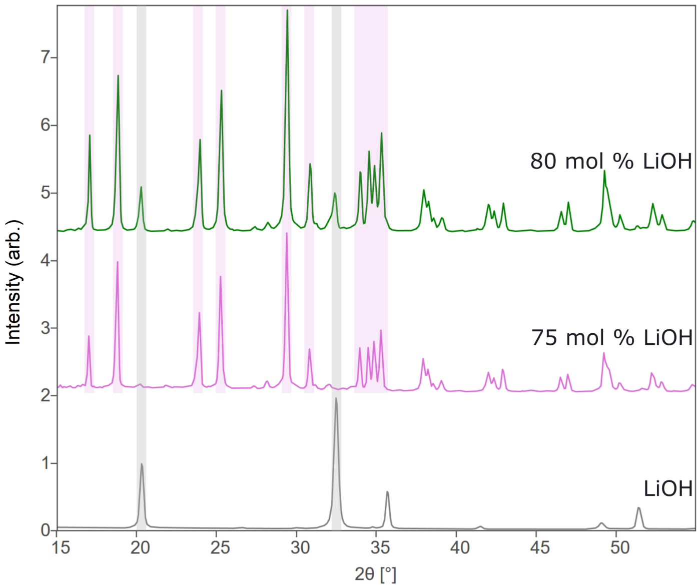

Figure S5: Comparison of 75 mol % LiOH and 80 mol % LiOH samples, quenched from 400 °C to room temperature and reheated to 250 °C for 12 hours, followed by quenching to room temperature again. 75 mol % LiOH samples exhibit the *Pmnm* phase, whereas an excess of LiOH is also present in the 80 mol % LiOH samples. Minor impurity peaks can be seen in both.

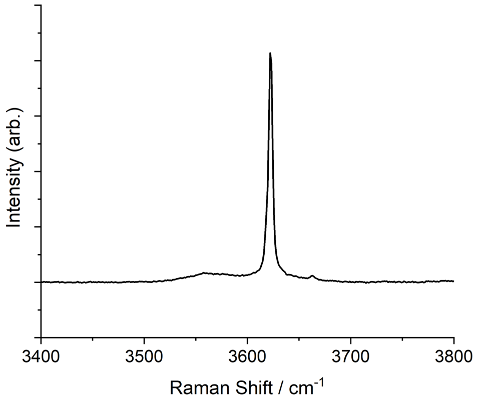

Figure S6: Raman spectroscopy of the -O-H bond stretching vibration region of room-temperature $Li_4(OH)_3Br$ after annealing at 250 °C. A strong signal at 3622 cm^{-1} arises from the $Li_4(OH)_3Br$ phase, with only minor impurity peaks, supporting the conclusion that the sample has a high phase purity.

Supporting Information Note 2 - Phase Diagram Considerations

Mahroug *et al.* used DSC peaks to propose a phase diagram, indicating phase transitions at $\sim 230\,°C$ and $\sim 280\,°C$ for compositions with 75 mol % LiOH or higher (Figure 1a) [9]. The inclusion of these phase fields implies polymorphs of $Li_4(OH)_3Br$ exist, although this is not something explicitly discussed in their study. Our findings, however, indicate that the phase transition at 230 °C does not belong on the phase diagram, as the DSC peak which provides the basis of its inclusion, is a result of non-equilibrium solidification. Specifically, the DSC peak arises upon heating when $Li_3(OH)_2Br$ forms from the $Li_4(OH)_3Br$-Li_2OHBr mixture found in furnace-cooled samples. Li_2OHBr is also present in furnace-cooled samples of 80 mol % LiOH samples (Figure S7), supporting this conclusion. Similarly, it can be expected that Mahroug's DSC peaks at 280 °C, used to define another phase field, are also a result of non-equilibrium processes. In this instance, the DSC peaks are associated with the melting of the $Li_3(OH)_2Br$ present in samples above 230 °C, which is expected to occur at 280 °C [2].

Nevertheless, we believe that further consideration may be required for the high-temperature portion of the phase diagram. Notably, diffraction patterns from furnace-cooled 75 mol % and 80 mol % LiOH samples cannot be fitted using solely the $Li_4(OH)_3Br$, Li_2OHBr and LiOH phases, due to unexplained intensity remaining. This may suggest that another phase field exists, or alternatively could be a further consequence of non-equilibrium solidification. It is more challenging to study the high-temperature polymorph region plotted between $\sim 280\,°C$ and $\sim 290\,°C$ due to the narrow temperature range available prior to melting. To mitigate impacts of non-equilibrium solidification, anneals of quenched 75 mol % and 80 mol % LiOH samples were carried out at 284 °C for 24 hours, followed by quenching to room temperature, to try and retain the indicated phase field to room temperature. The resulting diffraction patterns differed from those of samples annealed at 250 °C, indicating the possibility of a polymorph or another phase field in this region (Figure S7). Any additional phase transitions here, whether equilibrium or non-equilibrium processes, will also impact the observed melting enthalpy of $Li_4(OH)_3Br$. In the DSC profiles in Figure 4, it is possible to see a third, minor peak in addition to the 2 main peaks assigned to the melting of $Li_3(OH)_2Br$ and melting of $Li_4(OH)_3Br$. It is possible that this third peak corresponds to a phase transition associated with these findings. However, the narrow temperature range and slow solid-state kinetics suggest that the extent of the transformation occurring within the timescale of DSC heating measurements prior to melting is limited. Thus, the melting enthalpy reported in this work represents a close approximation to the true melting enthalpy of the *Pmnm* $Li_4(OH)_3Br$ phase. To understand the full impact of a potential polymorph, further investigation into the high-temperature portion of the phase diagram will be necessary.

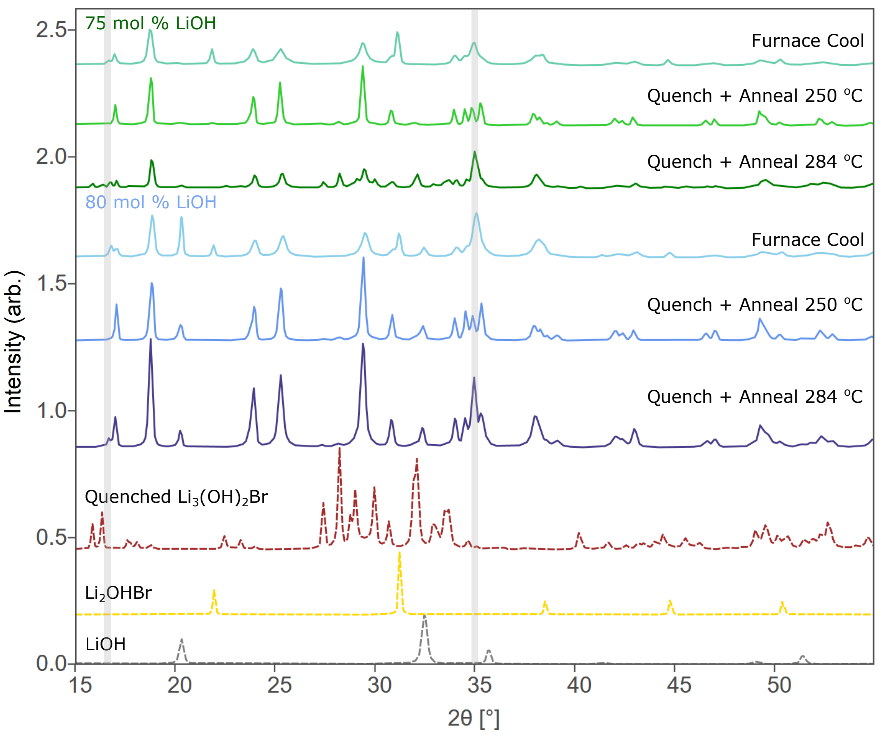

Figure S7: Comparison of XRD patterns for samples undergoing different heat treatments. Unexplained peaks (highlighted) observed in furnace-cooled samples may be a result of non-equilibrium solidification, however their presence in samples annealed at 284 °C indicates that another phase field may exist. Further investigation will be required to fully understand this region of the phase diagram.

Supporting Information Note 3 - Li-ion Dynamics

Aside from applications in TES, it is interesting to evaluate the lithium-ion dynamics in $Li_4(OH)_3Br$ in case of superionic conductivity which could make it of interest in solid electrolyte research, as with the closely-related compound Li_2OHBr [10–12]. To establish the ionic conductivity of $Li_4(OH)_3Br$, Electrochemical Impedance Spectroscopy (EIS) measurements were taken on cold-pressed pellets with Ni foil blocking electrodes in 10 °C intervals from 20 - 100 °C from 35 MHz to 0.1 Hz with a voltage amplitude of 10 mV. Despite conductivities of Li_2OHBr being easily obtained by this method at room temperature, no measurements were possible for $Li_4(OH)_3Br$ in this temperature range, suggesting a much lower ionic conductivity. To confirm this, molecular dynamics was performed on the crystal structure proposed in this work. In this, negligible Li-ion mobility is observed.

In 1981, Hartwig *et al.* reported the ionic conductivity of $Li_4(OH)_3Br$ to be comparable to that of the antiperovskite Li_2OHBr [13]. These early measurements were only possible at 200 °C, giving values of $5 \times 10^{-5} Scm^{-1}$ for both $Li_4(OH)_3Br$ and Li_2OHBr. No diffraction patterns were reported in the work by Hartwig, raising uncertainty as to what was being measured. Our findings indicate that *Pmnm* $Li_4(OH)_3Br$, reported here, is a much worse ionic conductor than Li_2OHBr, and should not be pursued as a candidate solid-state electrolyte. In addition, care should be taken to avoid the formation of $Li_4(OH)_3Br$ impurities in Li_2OHBr synthesis, for example by using an excess of LiOH as has been reported in the chloride homologue Li_2OHCl [14].

a

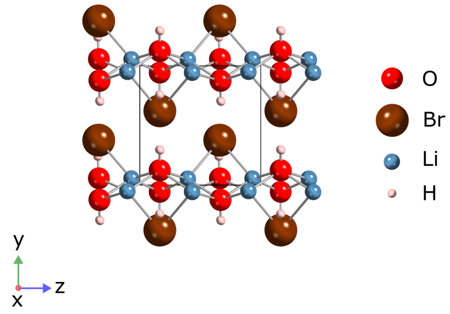

b

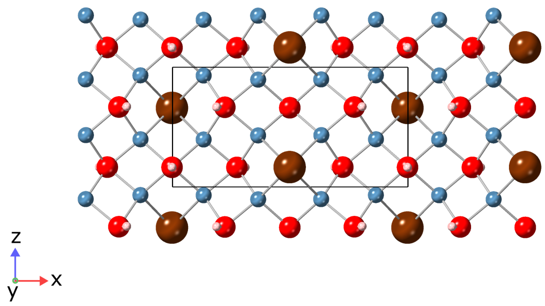

c

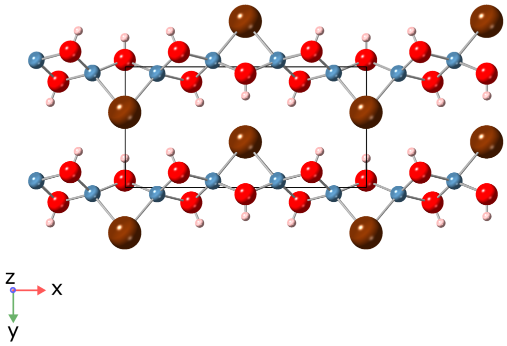

Figure S8: Computationally-predicted crystal structure for $Li_4(OH)_3Br$ found using AIRSS and optimisation, viewed along a) [100], b) [010] and c) [001]. Atomic positions are detailed in Table S3.

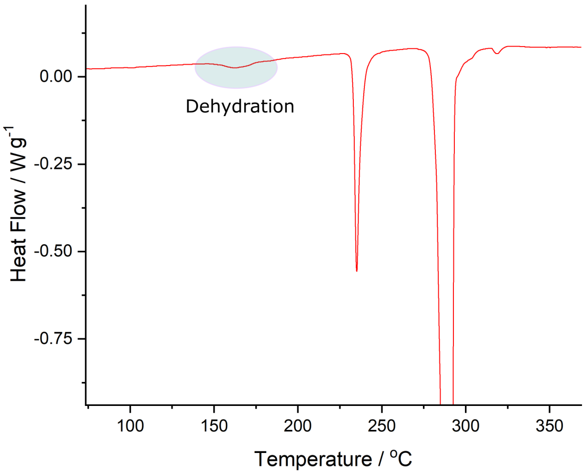

Figure S9: Magnification of DSC of the first heating cycle of hydrated $P2_1/m$ $Li_4(OH)_3Br$, showing a small peak corresponding to dehydration between 150 °C and 175 °C

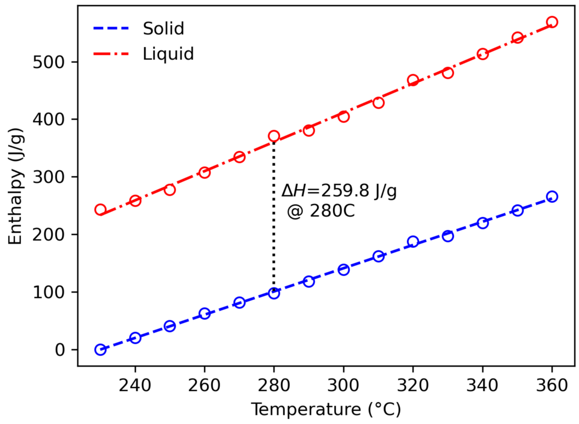

Figure S10: Plot used to theoretically estimate the enthalpy of melting of $Li_4(OH)_3Br$ from the computationally-predicted crystal structure. A value of 259.8 J g^{-1} is found from the difference between the solid and liquid lines at 280 °C.

Table S1: Unit cell parameters for the $P2_1/m$ structure of air-exposed $Li_4(OH)_3Br$ at room temperature from the Pawley fit in Figure 2c

Unit Cell	
a (Å)	5.4594(3)
b (Å)	7.5922(3)
c (Å)	6.5090(3)
β (°)	93.841(2)
V (Å^3)	269.18(2)

Table S2: Unit cell parameters determined from a Pawley fit for the $Pmnm$ structure of $Li_4(OH)_3Br$ at 250 °C.

Unit Cell	
a (Å)	10.4099(3)
b (Å)	5.30384(15)
c (Å)	5.27776(13)
V (Å^3)	291.399(14)

Table S3: Crystallographic parameters from the computationally-determined $Li_4(OH)_3Br$ crystal structure, shown in Figure S8

a (Å)	10.585518		
b (Å)	5.281199		
c (Å)	5.370332		
V (Å^3)	300.224241		
Atom	x	y	z
Br1	-0.002293	0.376336	0.660738
Br2	0.497465	0.622271	0.165855
Li1	0.363473	0.943347	0.430843
Li2	0.132275	0.055429	-0.068900
Li3	0.863662	0.052760	-0.067498
Li4	0.632133	0.941236	0.433144
Li5	0.132208	0.054273	0.396716
Li6	0.363737	0.949365	-0.103164
Li7	0.631353	0.948983	-0.101322
Li8	0.864054	0.049794	0.398415
O1	0.222310	0.877160	0.665833
O2	0.273436	0.124696	0.163587
O3	0.723143	0.122543	0.165993
O4	0.772337	0.873474	0.667914
O5	-0.001979	0.942669	0.164455
O6	0.498076	0.056167	0.662108
H1	0.186982	0.705983	0.668619
H2	0.308211	0.296109	0.163812
H3	0.689276	0.293827	0.167676
H4	0.805416	0.701549	0.671058
H5	-0.001788	0.758156	0.164731
H6	0.498654	0.240542	0.653282

Table S4: Crystallographic parameters for the $Pmnm$ structure of $Li_4(OH)_3Br$ *in-situ* at 250 °C, from the fit shown in Figure S3. Refined parameters are indicated with errors in brackets.

a (Å)	10.41180(17)					
b (Å)	5.30463(7)					
c (Å)	5.27890(7)					
V (Å^3)	291.557(7)					
Atom	Wyckoff position	x	y	z	Occupancy	U_{iso} (Å^2)
Br1	2b	0.25	0.3675(4)	0.75	1	0.006(2)
O1	4f	-0.02508	0.12415	0.25	1	0.006(2)
O2	2a	0.25	-0.05675	0.25	1	0.006(2)
Li1	8g	0.61587	-0.053665	0.5171	1	0.006(2)
H1	4f	-0.05934	0.2956	0.25	1	0.006(2)
H2	2a	0.25	0.75881	0.25	1	0.006(2)

Table S5: Unit cell parameters for the $P6_3/mmc$ structure of $Li_3(OH)_2Br$ [2] also present *in-situ* at 250 °C, from the fit shown in Figure S3.

Unit Cell	
$a = b$ (Å)	6.55948(6)
c (Å)	10.72619(14)
V (Å^3)	399.682(9)

References

[1] Alan A Coelho. Topas and topas-academic: an optimization program integrating computer algebra and crystallographic objects written in c++. *J. Appl. Crystallogr*, 51:210–218, 2 2018. doi: 10.1107/S1600576718000183.

[2] Emily Milan, James A. Quirk, Kenjiro Hashi, John Cattermull, Andrew L. Goodwin, James Dawson, and Mauro Pasta. Filling the gaps in the libr-lioh phase diagram: a study on the high-temperature li3(oh)2br phase. *ChemRxiv*, 1 2025. doi: 10.26434/chemrxiv-2025-m7mv6-v2. This content is a preprint and has not been peer-reviewed.

[3] Chris J. Pickard and R. J. Needs. Ab initio random structure searching. *J. Phys.: Condens. Matter*, 23(5):053201, 2011. ISSN 0953-8984. doi: 10.1088/0953-8984/23/5/053201.

[4] Stefan Grimme, Jens Antony, Stephan Ehrlich, and Helge Krieg. A consistent and accurate ab initio parametrization of density functional dispersion correction (DFT-D) for the 94 elements H-Pu. *J. Chem. Phys*, 132(15):154104, 2010. ISSN 0021-9606. doi: 10.1063/1.3382344.

[5] So Takamoto, Chikashi Shinagawa, Daisuke Motoki, Kosuke Nakago, Wenwen Li, Iori Kurata, Taku Watanabe, Yoshihiro Yayama, Hiroki Iriguchi, Yusuke Asano, Tasuku Onodera, Takafumi Ishii, Takao Kudo, Hideki Ono, Ryohto Sawada, Ryuichiro Ishitani, Marc Ong, Taiki Yamaguchi, Toshiki Kataoka, Akihide Hayashi, and Takeshi Ibuka. Towards universal neural network potential for material discovery applicable to arbitrary combination of 45 elements. *Nature Communications*, 13: 2991–3001, 2022.

[6] Shyue Ping Ong, William Davidson Richards, Anubhav Jain, Geoffroy Hautier, Michael Kocher, Shreyas Cholia, Dan Gunter, Vincent L. Chevrier, Kristin A. Persson, and Gerbrand Ceder. Python Materials Genomics (pymatgen): A robust, open-source python library for materials analysis. *Comput. Mater. Sci*, 68:314–319, 2013. ISSN 0927-0256. doi: 10.1016/j.commatsci.2012.10.028.

[7] Ask Hjorth Larsen, Jens Jørgen Mortensen, Jakob Blomqvist, Ivano E. Castelli, Rune Christensen, Marcin Dułak, Jesper Friis, Michael N. Groves, Bjørk Hammer, Cory Hargus, Eric D. Hermes, Paul C. Jennings, Peter Bjerre Jensen, James Kermode, John R. Kitchin, Esben Leonhard Kolsbjerg, Joseph Kubal, Kristen Kaasbjerg, Steen Lysgaard, Jón Bergmann Maronsson, Tristan Maxson, Thomas Olsen, Lars Pastewka, Andrew Peterson, Carsten Rostgaard, Jakob Schiøtz, Ole Schütt,

Mikkel Strange, Kristian S. Thygesen, Tejs Vegge, Lasse Vilhelmsen, Michael Walter, Zhenhua Zeng, and Karsten W. Jacobsen. The atomic simulation environment—a Python library for working with atoms. *J. Phys.: Condens. Matter*, 29(27):273002, 2017. ISSN 0953-8984. doi: 10.1088/1361-648X/aa680e.

[8] Cecilia M. S. Alvares, Guillaume Deffrennes, Alexander Pisch, and Noël Jakse. Thermodynamics and structural properties of CaO: A molecular dynamics simulation study. *J. Chem. Phys*, 152(8): 084503, 2020. ISSN 0021-9606. doi: 10.1063/1.5141841.

[9] Imane Mahroug, Stefania Doppiu, Jean Luc Dauvergne, Jean Toutain, and Elena Palomo del Barrio. Extended investigation of lioh–libr binary system for high-temperature thermal energy storage applications. *J. Therm. Anal. Calorim*, 147:12455–12465, 11 2022. ISSN 15882926. doi: 10.1007/s10973-022-11468-4.

[10] Jingfeng Zheng, Brian Perry, and Yiying Wu. Antiperovskite superionic conductors: A critical review. *ACS Mater. Au*, 1:92–106, 11 2021. ISSN 2694-2461. doi: 10.1021/acsmaterialsau.1c00026.

[11] James A. Dawson, Theodosios Famprikis, and Karen E. Johnston. Anti-perovskites for solid-state batteries: recent developments, current challenges and future prospects. *J. Mater. Chem. A*, 9: 18746–18772, 2021. ISSN 2050-7488. doi: 10.1039/d1ta03680g.

[12] Wei Xia, Yang Zhao, Feipeng Zhao, Keegan Adair, Ruo Zhao, Shuai Li, Ruqiang Zou, Yusheng Zhao, and Xueliang Sun. Antiperovskite electrolytes for solid-state batteries. *Chem. Rev*, 122:3763–3819, 2 2022. ISSN 15206890. doi: 10.1021/acs.chemrev.1c00594.

[13] P Hartwig, A Rabenau, and W Weppner. Lithium hydroxide halides: Phase equilibria and ionic conductivities. *J. Less Common Met.*, 78:227–233, 1981.

[14] Fei Wang, Hayden A. Evans, Kwangnam Kim, Liang Yin, Yiliang Li, Ping Chun Tsai, Jue Liu, Saul H. Lapidus, Craig M. Brown, Donald J. Siegel, and Yet Ming Chiang. Dynamics of hydroxyl anions promotes lithium ion conduction in antiperovskite li2ohcl. *Chem. Mater*, 32:8481–8491, 10 2020. ISSN 15205002. doi: 10.1021/acs.chemmater.0c02602.
